# Nano- to microscale three-dimensional morphology relevant to transport properties in reactive porous composite paint films

**DOI:** 10.1038/s41598-020-75040-6

**Published:** 2020-10-27

**Authors:** Xiaoyang Liu, Valeria Di Tullio, Yu-Chung Lin, Vincent De Andrade, Chonghang Zhao, Cheng-Hung Lin, Molly Wagner, Nicholas Zumbulyadis, Cecil Dybowski, Silvia A. Centeno, Yu-chen Karen Chen-Wiegart

**Affiliations:** 1 Department of Materials Science and Chemical Engineering, Stony Brook University, Stony Brook, NY 11794 USA; 2 Department of Scientific Research, The Metropolitan Museum of Art, New York, NY 10028 USA; 3 “Segre-Capitani” Magnetic Resonance Laboratory, Istituto Per I Sistemi Biologi, (ISB) CNR, CNR Area Della Ricerca di Roma 1, Via Salaria Km 29, 300, 00015 Monterotondo, Rome, Italy; 4 Advanced Photon Source, Argonne National Laboratory, Argonne, IL 60439 USA; 5 Department of Chemistry and Biochemistry, University of Delaware, Newark, DE 19716 USA; 6 Independent Researcher, Rochester, NY 14613 USA; 7 National Synchrotron Light Source II, Brookhaven National Laboratory, Upton, NY 11973 USA

**Keywords:** Chemical physics, Composites

## Abstract

The quantitative evaluation of the three-dimensional (3D) morphology of porous composite materials is important for understanding mass transport phenomena, which further impact their functionalities and durability. Reactive porous paint materials are composites in nature and widely used in arts and technological applications. In artistic oil paintings, ambient moisture and water and organic solvents used in conservation treatments are known to trigger multiple physical and chemical degradation processes; however, there is no complete physical model that can quantitatively describe their transport in the paint films. In the present study, model oil paints with lead white (2PbCO_3_·Pb(OH)_2_) and zinc white (ZnO) pigments, which are frequently found in artistic oil paintings and are associated with the widespread heavy metal soap deterioration, were studied using synchrotron X-ray nano-tomography and unilateral nuclear magnetic resonance. This study aims to establish a relationship among the paints’ compositions, the 3D morphological properties and degradation. This connection is crucial for establishing reliable models that can predict transport properties of solvents used in conservation treatments and of species involved in deterioration reactions, such as soap formation.

## Introduction

Works of art are typically hybrid materials consisting of inorganic and/or organic components structured in complex ways. The nature of these materials used in works of art resembles many other functional composite materials. In traditional artistic oil paintings, inorganic and/or organic pigments are mixed with a drying oil binder, usually applied in multiple layers over a substrate, such as canvas, wood, or metal, with protective organic coatings on top. Deterioration processes of the oil paint films often involve interactions at the interfaces of these components, with degradations in one triggering subsequent chemical reactions and physical changes in another.

Soap formation in oil paintings is one of the most pervasive forms of deterioration which occurs when saturated fatty acids, that result from the hydrolisis of glycerides in the oil binding medium, react with heavy metals in the pigments, driers, or other addtitives to produce carboxylates, also called soaps. Among the pigments most frequently affected by saponification are lead- and zinc-based, such as lead white, lead tin yellow, and zinc white. This process may cause flaking, delamination, increased transparency, and the formation of protrusions, crater-like holes, and surface crusts^1–7^. High temperature and relative humidity, as well as aqueous solutions and organic solvents used in conservation treatments, are important factors known to trigger soap formation^7–10^, in addition to causing other physical and chemical degradation processes, such as paint swelling, the deformation of canvas supports, and pigment color changes^11–15^. Baij * et al.* reported that solvent flow enhances soap crystallization in model oil paint systems by displacing reactive molecules, such as palmitic acid^8^, and that the amount of water also has an effect on the rate of soaps crystallization. We hypothesize that heavy metal cations from pigments or additives and fatty acid anions or aggregates migrate through the paint matrix assisted by the diffusion of water or solvents via interconnected channels and pores of various sizes, ultimately forming soaps. Therefore, the, three-dimensional (3D) morphological parameters such as pore size, connectivity, and tortuosity will affect the diffusion of water and organic molecules in the films, and thus directly impact the deterioration.

A wide range of methods have been used to characterize the reactive species, free fatty acids and pigments, and products of soap deterioration in both microsamples removed from artistic paintings and model paint samples (See, for example^1,3,5,6,16–22^). Boon *et al.* utilized neutron radiography on a mesoscopic level to study water uptake by model samples of painting canvas supports and ground preparations. They reported that the canvas cellulose fibers and the glue sizing have a much stronger water uptake than the chalk ground layer bound in glue, and that the uptake rate is not uniform throughout the thickness of the sized canvas^23^.

Nuclear magnetic resonance (NMR) allows the measurement of the longitudinal and transverse relaxation times, T_1_ and T_2,_ two important parameters can provide critical information about the physiochemical properties of cross-link polymers and water-saturated media and are used often to characterize geologic materials^24–26^.

Unilateral NMR permits the open porosity available to the diffusion of water and solvents in different materials to be determined^27,28^. In this article, with ‘open porosity’ we refer to the void fraction or ‘empty’ spaces that may be filled by water or organic solvents in contact with the paint film. The most common NMR methods to probe the structure of porous media are based on the measurement of relaxation times and the diffusion coefficient of water inside the porous systems. Relaxation times of fluids confined in porous media are strictly related to the geometry of the structure, as water in small pores relaxes rapidly, whereas water in large pores relaxes more slowly^29,30^. In many cases the properties of porous systems may be spatially resolved by means of magnetic resonance imaging (MRI)^31^. The development of unilateral NMR sensors, which allow one to study arbitrarily sized objects non-invasively by combining open magnets and surface RF coils to generate a sensitive volume external to the sensor and inside the object under investigation, was a breakthrough for the application of NMR to study materials relevant to cultural heritage applications^32,33^. Although the magnetic field of this sensor is inhomogeneous, it is possible to measure NMR parameters such as proton density, relaxation times, self-diffusion coefficients, and even to collect correlation maps^34^. Due to its capability to measure the hydrogen content as a function of the depth of the measurement, unilateral NMR is a very powerful method for scanning layers composed by organic substances as well as for measuring the water content absorbed by porous materials^35–38^.

X-ray tomography is a powerful tool for characterizing the 3D morphology of paints. For example, in a sample of a ground preparation layer composed of calcium carbonate bound by a proteinaceous medium taken from a nineteenth century painting, Gervais *et al.* calculated a total porosity of about 15% from X-ray micro-tomography measurements using a 370 nm pixel size^39^. Ma *et al.*^22^ used photothermal induced resonance in combination with IR microspectroscopy and X-ray micro-tomography to study the distribution of metal carboxylayes in a naturally aged, 23-year old Zn white oil paint containing aluminum stearate as an additive. These studies highlight the capability of X-ray micro-tomography to reveal the internal 3D structure of paints. The recent development of X-ray nano-tomography with full-field transmission X-ray microscopy (TXM) provides tens of nanometer spatial resolution with a field of view of tens of microns, making it possible to study oil paints with an even higher spatial resolution^40–43^.

While analyses of samples removed from works of art give in-depth information about the 3D morphological parameters^22,39^, the characterization of model samples prepared under controlled conditions with varying paint compositions, including different pigments, pigment-to-oil ratios and additives, permit to better understand the roles of these conditions on the morphological features that affect transport. In the present study, model paints with two pigments frequently associated with heavy metal soap deterioration in oil paintings were studied, namely lead white (2PbCO_3_ $\bullet$ Pb(OH)_2_) and zinc white (ZnO). While lead white has been reported to form soaps in old master, modern, and contemporary works (see, for example^2–4,44,45^), zinc white, which emerged during the end of the eighteenth century^46^, is commonly associated with the phenomenon in paintings dating from the middle of the nineteenth century on^1,5,45,47^. Aluminum (Al) stearate was frequently added to twentieth century commercial paint formulations to prevent the separation of the pigment from the oil; however, it has been shown that the compound is a source of free fatty acids and that it may promote saponification^1,5,47^. Therefore, Al stearate was added to a set of the zinc white oil paint samples prepared for this study.

The model paint samples were characterized by novel synchrotron X-ray nano-tomography and NMR methods. Synchrotron X-ray nano-tomography enables a direct visualization of the pigment particles and Al stearate agglomerates in the polymerized oil network^48^. Unilateral NMR was used to measure the hydrogen content as a function of the depth in the paint films to obtain the water content absorbed by the pores^36–38^. By quantifying the volume fraction and feature size distribution of the particles, the tortuosity, and open porosity connected to the diffusion of water using these techniques, the potential effect of these properties on mass transport and the possible path for mobile materials were evaluated. This understanding is crucial to provide guidance for conservation treatments and insights into deterioration mechanisms. The knowledge of how nano- to microscale 3D morphology relevant to transport properties varies as a function of materials constituents, is also critical for a wider range of composite materials.

## Experimental section

### Sample preparation and preliminary characterization

**Figure 1:**
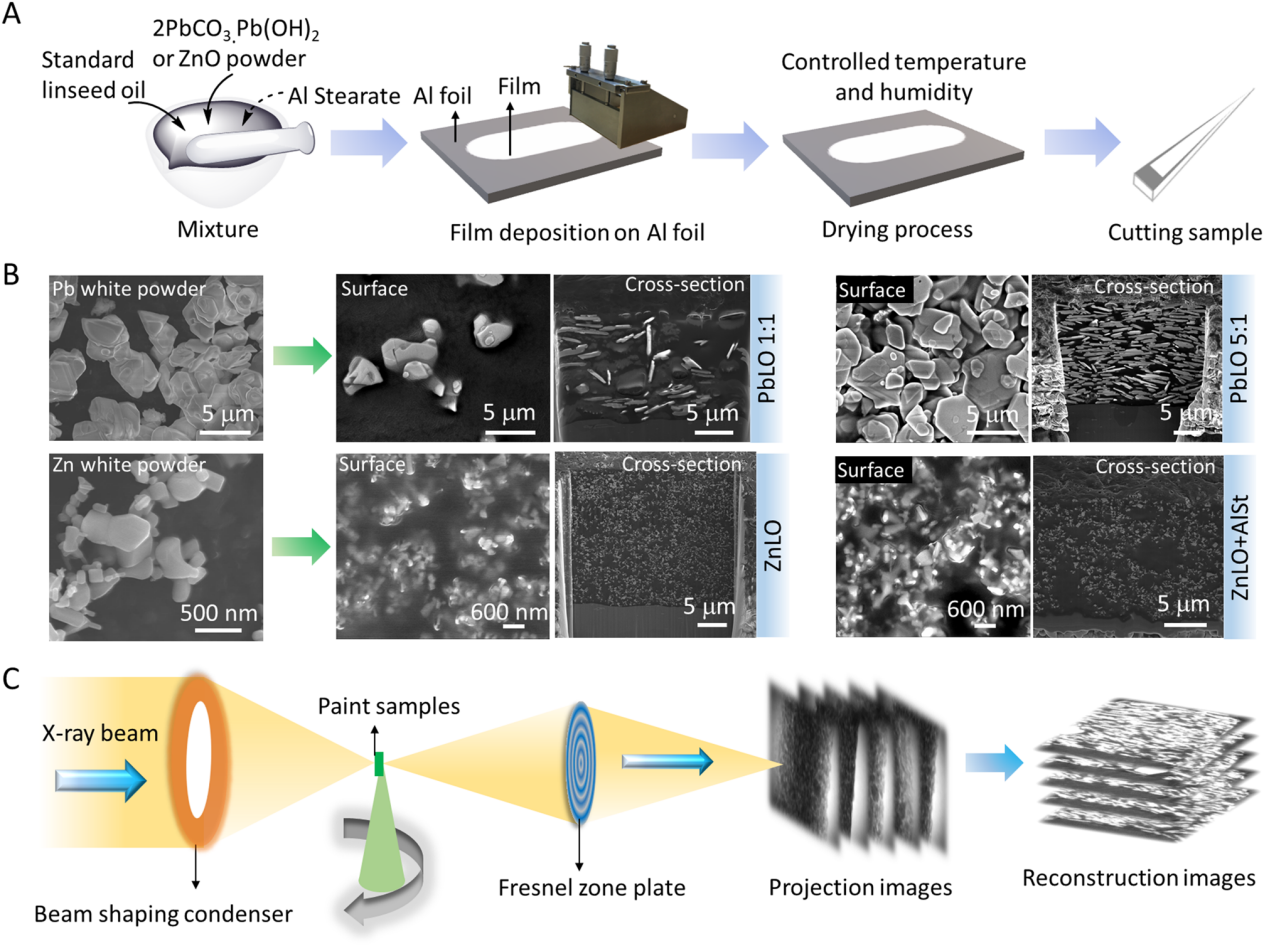
(**A**) Paint sample preparation process. (**B**) Surface morphology of Pb white and Zn white powders, and surface and cross-section morphology of the oil paints imaged: PbLO 1:1, PbLO 5:1, and ZnLO without Al stearate and with 10 wt.% Al stearate (ZnLO + AlSt). (**C**) X-ray nano-tomography set up and reconstruction workflow.

Pb white (2PbCO_3_ $\bullet$ Pb(OH)_2_) and Al stearate (Al(OH)_2_C_18_H_35_O_2_) were purchased from Sigma-Aldrich. Zn white (ZnO) was purchased from Alfa Aesar. Pb white was mixed with standard linseed oil (LO, Kremer Pigments Inc.) with pigment-to-oil weight ratios of 1:1 and 5:1. Zn white was mixed with standard LO with a 1:1 pigment-to-oil weight ratio, with and without 10 wt.% Al stearate. The paint samples with the two pigments are labeled PbLO and ZnLO, respectively. After fully mixing, the paints were applied on Al foil, as 90 μm thick layers for PbLO and as 45 μm layers for ZnLO, using a film applicator, at room temperature (Fig. 1A). As mentioned in the main text, the resulting wet films were thinner than the applicator gap setting.

Additional samples were made by applying the paints on canvas supports. The PbLO 1:1 paint was deposited on a canvas support prepared with a calcite (CaCO_3_) layer bound in glue applied by brush, and the ZnLO 1:1 and 3:1 paints were applied directly on the canvas substrates. For all the sets of paints on canvas, the pigmented paint film was applied using a film applicator with an initial (wet) thickness of about 200 µm. For curing, all the samples were stored at room temperature under a humidity-controlled environment (*ca.* 52%RH) for six months.

The relative changes in the film thicknesses upon curing were determined by unilateral NMR. For this task, paint films with similar compositions as those listed in Table 1 were applied on glass slides with an approximate 200 μm initial (wet) thickness. For the X-ray nano-tomography measurements, the samples were cut with a scalpel and mounted onto tomographic sample mounting pins. To make it compatible with the field of view of the TXM and to ensure sufficient X-ray transmission, the tip of the sample was < 50 μm.

**Table 1:** Conditions used in the preparation of the samples used in this study, including different pigments, pigment-to-linseed oil (LO) weight ratios, Al stearate concentration, and film thicknesses.

Sample ID	Pigment	Pigment to linseed oil (LO) weight ratio	Al stearate (wt.%)
PbLO 1:1	Lead white	1:1	0
PbLO 5:1	Lead white	5:1	0
ZnLO	Zinc white	1:1	0
ZnLO + AlSt	Zinc white	1:1	10
PbLO 1:1 + chalk + canvas	Lead white	1:1	0
Zn 1:1 − canvas	Zinc white	1:1	0
Zn 3:1 − canvas	Zinc white	1:1	0

### Surface and cross-section morphology

To characterize the surface morphology of the pigments and Al stearate powders, and the surface and cross-section morphology of the oil paint samples (Fig. 1B), a Hitachi S4800 SEM with a 10.0 kV accelerating voltage was used. FIB-SEM (FEI Helios) was utilized to mill the paint samples at 30.0 keV with decreasing currents in the following sequence: 21, 9.2, 6.5, 2.8 and 0.92 nA, in order to observe the entire cross-section profile, from the surface to the substrate.

### Synchrotron X-ray nano-tomography

X-ray nano-tomography measurements were carried out at TXM beamline 32-ID-C, Advanced Photon Source (APS), Argonne National Laboratory (ANL)^43^. A beam shaping condenser was used to illuminate the sample and a Fresnel zone plate (60 nm) was used as an objective lens (Fig. 1C). The monochromatic beam energy was set at 9.76 and 8 keV for the ZnLO and PbLO samples, respectively. For each sample, 1201 projections over 180° rotation were collected with a lens-coupled 2D area detector. The field of view of the TXM was 64 µm × 54 µm for 8 keV with a pixel size of 26.16 nm, and the pixel size at 9.76 keV was 31.70 nm.

To reconstruct the 3D structures, the Gridrec algorithm with TomoPy was used^49,50^. Thresholding segmentation and thickness comparison were carried out on the reconstructed images of Pb-white paints in Image J^51^. For the Zn white samples, because the gray-scale distribution of the oil and pigment phases overlap in the histogram, the segmentation was based on visually finding possible minimum, mean, and maximum threshold values to account for the variation due to thresholding. The assessment on the effect of thresholding segmentation is presented in Supplementary Information Figures S1-5 and Supplementary Information Tables S1 and S2. The mean threshold value was used to segment the reconstructed images for the quantitative analysis of the morphological parameters. The thickness of cured paint films was measured by calculating the full-width-half-maximum of the X-ray attenuation *vs.* distance from the surface to the substrate of the paint films; the process was repeated at three different locations of the image to obtain the average thickness and variation. The visual comparison, specific surface area analysis, phase connectivity and 3D visualization were conducted in a commercial software AVIZO (v.9.3 FEI) and open-source software TOMVIZ software^52^. Further morphological analysis, including phase volume fraction, particle size distribution, and tortuosity quantification, were conducted using customized codes developed in-house with details described in prior articles^53–55^.

### Unilateral NMR

^1^H depth profiles and spin–spin relaxation times (T_*2eff*_), were measured at 0.36 T (proton Larmor frequency of 18 MHz) with an NMR-MOUSE (Mobile Universal Surface Explorer) instrument consisting of an electronic unit manufactured by Bruker Biospin interfaced with a single-sided sensor ACT by RWTH Aachen University, Aachen, Germany^35^. This sensor generates an inhomogeneous magnetic field with an extremely uniform gradient to resolve the near surface structure of arbitrarily large objects. ^1^H depth profiles were obtained by averaging the amplitude of the first eight points of an echo train of 32 echoes with an echo time (*2τ*) of 41 µs, using the Carr-Purcell-Meiboom-Gill (CPMG) pulse sequence^56^. The nominal spatial resolution was 50 µm.

Since the spatial resolution of unilateral NMR is lower compared to TXM, four samples with similar compositions as those listed in Table 1, but with larger thickness of ~ 200 μm, were applied on glass slides for the NMR measurements. The NMR-MOUSE sensor was re-positioned in steps of 50 and 100 µm to cover the desired spatial range, from the surface of the sample to a depth of about 600 µm, a position within the glass substrate with a zero ^1^H NMR signal.

Effective spin–spin relaxation times, T_*2eff*_, were measured with a CPMG pulse sequence. 1024 echoes were recorded in the center of the sample with an echo time of 41 µs. Due to the presence of an inhomogeneous magnetic field, the observed spin–spin relaxation time, T_*2eff*_, was always shorter than the T_2_ that would be measured in a homogeneous magnetic field. Consequently, the quantity measured by unilateral NMR is usually called effective spin–spin or transverse relaxation time, *T*_*2eff*_. The decay of the transverse magnetization was fit using an inverse Laplace transform (ILT) of the data to obtain the distribution of T_*2eff*_.The ILT is particularly useful when the signal is characterized by a multi-exponential decay. The ILT of the data was obtained by using the Uniform Penalty (UPEN) algorithm^57^. Data was first normalized by determining the mean offset and subsequently processed with the UPEN algorithm.

The effective free volume available for water diffusion was evaluated from the CPMG decays, which allows one to obtain the value of the proton spin density at t = 0 by fitting the experimental decay. To calculate the open porosity filled by water throughout the film, the samples were placed in contact with a water source following a procedure similar to that reported for measuring water capillary absorption in other porous materials^58,59^. Small modifications to this process were made to control the water absorption, *i.e.* the sample was covered with several layers of filter paper saturated with water on a glass substrate and was wrapped in a plastic film to slow down the evaporation during the measurements. After 72 h of water absorption, the T_*2eff*_ was measured in the center of the film layer. The experimental data, ^1^H NMR normalized signal intensity (*Y*) was fit to the following equation:

$$Y={C}_{0}+\sum_{i=1}^{n}{M}_{0i} {e}^{(-2\tau /{T}_{2i})},$$

where *n* is the number of components of the CPMG decay of the magnetization, *2τ* is the echo time, *M*_*0i*_ and *T*_*2i*_ are the weight and the spin–spin relaxation time of the i-^th^ component, respectively, and *C*_*0*_ is the offset value, which accounts for the noise of the measurement. The values obtained are reported in the Supporting Information file (Table S4).

## Results and discussion

### Distribution and mobility of proton domains and open porosity available for the diffusion of water in lead white and zinc white oil paints as determined by unilateral NMR

**Figure 2:**
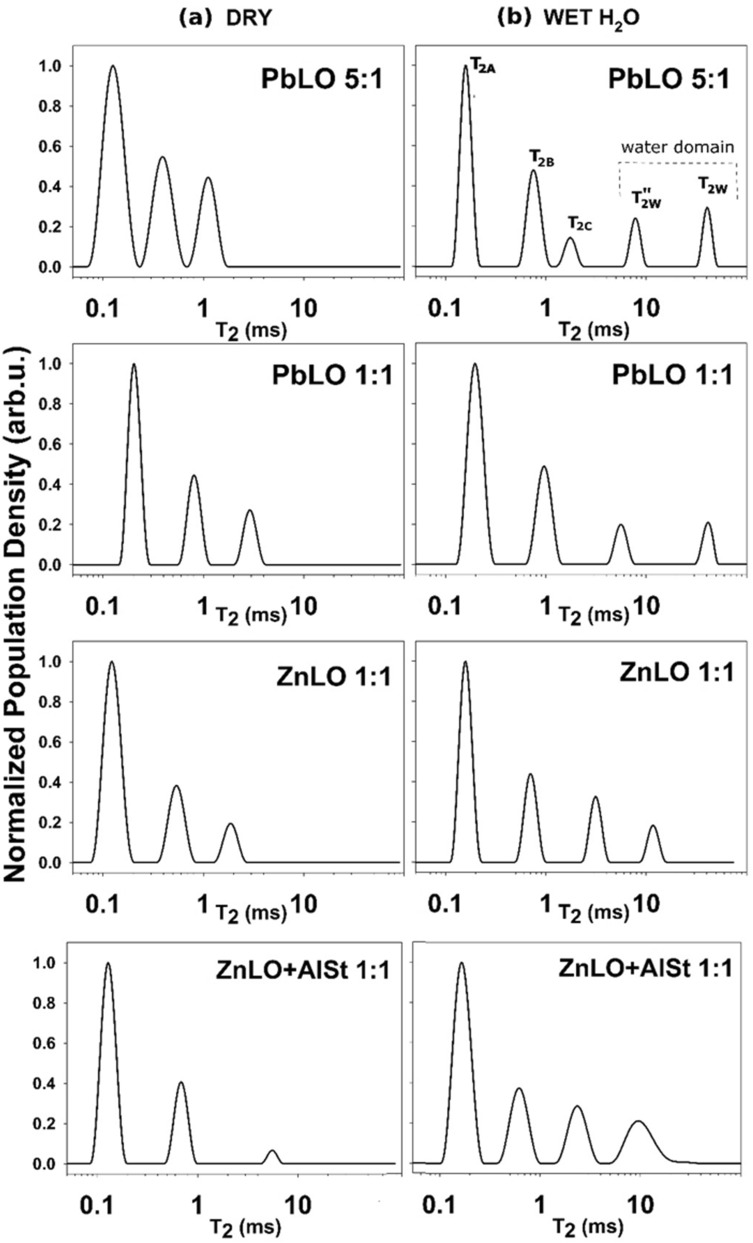
Distribution of T_2*eff*_ in (**a**) dry paint samples and (**b**) after a 72 h hour water absorption period. The population density reported has been normalized.

To evaluate how the water absorption and the open porosity of oil paints can be effected by zinc white and lead white pigments in different concentrations, and by the presence of the aluminum stearate additive, we analyzed the transverse relaxation times in the cured paint samples before and after water absorption. In all the dry samples, three components of T_*2eff*_ were observed (Fig. 2a). The short component (T_2A_ ~ 0.13–0.2 ms) that affects the majority of the protons arises from protons in the relatively rigid framework of the polymeric oil network; the intermediate component (T_2B_ ~ 0.5–0.8 ms) reflects protons in the polymer chain portions that have some relative motional freedom (e.g. methyl groups at the ends of fatty chains); and the third component (T_2C_ ~ 2–4 ms) is due to protons in residual unsaturated fatty acids not fully cross linked or saturated fatty acids not linked to the polymeric network (e.g. palmitic and stearic acid) that have a higher mobility that those in the rigid matrix. Interestingly, all paint samples with a higher amount of linseed oil showed a slight shift of T_2*eff*_ towards higher values indicating a less rigid or less cross-linked polymeric film. This result is not surprising, since as the concentration of lead white increases, its drying action increases as well^60^.

After 72 h of water absorption, the distributions of T_*2eff*_ have changed (Fig. 2b). All wet samples show an additional T_2w_ long component (with a value > 10 ms) that arises from protons in a relatively free water domain. Two macro-domains of proton mobility can be identified: the first domain, with T_*2eff*_ from 0.1 to 5 ms, reflects the mobility of protons in the polymerized oil with the same three components found in dry samples; the second domain, with T_*2eff*_ > 10 ms, reflects the proton mobility of the water molecules.

In all samples, long and intermediate T_*2eff*_ of protons in the polymerized oil are unaffected by water absorption. On the contrary, T_2C_, that arises from protons in not cross-linked fatty acids, shows a different behavior depending on the type and concentration of pigment and on the presence of the additive. With low pigment to oil ratios, both PbLO 1:1 and ZnLO 1:1 show an increased mobility of protons in the not cross-linked fatty acids in the presence of water (T_2C_ increases). The presence of mobile domains in polymerized oil has been previously reported and have been suggested to have an important plasticizing role^61,62^. In the presence of Al stearate, ZnLO + AlSt 1:1 shows an increase in the proton population density of T_2C_. Since Al stearate is not part of the oil cross-linked network, it may experience a higher degree of mobility in the presence of water.

Finally, in PbLO 5:1, two types of water, one free (T_2w_) and one bound (Tʺ_2w_), were observed. These results are similar to those reported in our previous study^27^. Also, in this case, the appearance of a second domain of water may be due to the formation of hydrated ionic group clusters. Interestingly, the presence of bound water is not observed at low concentrations of lead white. The formation of these hydrated ionic groups in lead-based paints may be triggered by the high amount of pigment and of absorbed water. Furthermore, it was found that protons of absorbed water have a lower degree of mobility in oil paints containing zinc white (T_*2eff*_ ~ 20 ms) than in paints with lead white (T_*2eff*_ ~ 46 ms) with the same pigment to oil ratio (1:1). The T_*2eff*_ of water may be affected by the different morphology of the porous network like in saturated rocks where a smaller pore size correlates with smaller values of T_*2eff*_. Nevertheless, in oil paints it is not possible to neglect the possible interactions of water with the polymeric oil network that might influence the relaxation of water. As a consequence, in paints, the distribution of T_*2eff*_ cannot be directly associated with the distribution of porosity as is in stones and rocks^30^.

Carr-Purcell-Meiboom-Gill (CPMG) decay and can be used to calculate the open porosity of the samples. To calculate the effective open porosity, the amplitude of the spin proton density of water, *M*_*0w*_, was extrapolated and the contributions of the protons in the polymerized oil domain were neglected (for details, see the Supporting Information file). The effective open porosity was calculated by normalizing the value of the water absorbed with respect to the value obtained for bulk water (i.e., 100% of porosity)^58,59^.

The M_0w_ value was normalized to the amplitude measured on bulk water *M*_*0b*_, which is equivalent to an effective porosity of 100%:

1 $$ \varTheta _{NMR} = \left( {M_{{0w}} /M_{{0b}} } \right) \times 100 $$

Using Eq. (1), values of the open porosity *Θ*_*NMR*_ available to water were calculated to be 2.0 and 5.0% for the PbLO 1:1 and 5:1 samples, respectively. For the ZnLO samples with and without Al stearate, the values obtained were 1.7 ± 0.5 and 7.2% ± 1.0, respectively (Table 2).

**Table 2:** Values of the open porosity available for water diffusion in the dry samples as determined by unilateral NMR.*Uncertainty calculated with a variation of 10% on M_0w_ (see Supporting Information file).

Sample ID	Pigment to oil weight ratio	Al stearate (wt.%)	Open porosity *Θ*_NMR_ ± Δ*Θ*_NMR_* (%)
PbLO 1:1	1:1	0	2.0 ± 0.5
PbLO 5:1	5:1	0	5.0 ± 1.0
ZnLO	1:1	0	1.7 ± 0.5
ZnLO + AlSt	1:1	10	7.2 ± 1.0

### Effect of the pigment to oil weight ratio on the 3D morphological properties of lead white oil paints

**Figure 3:**
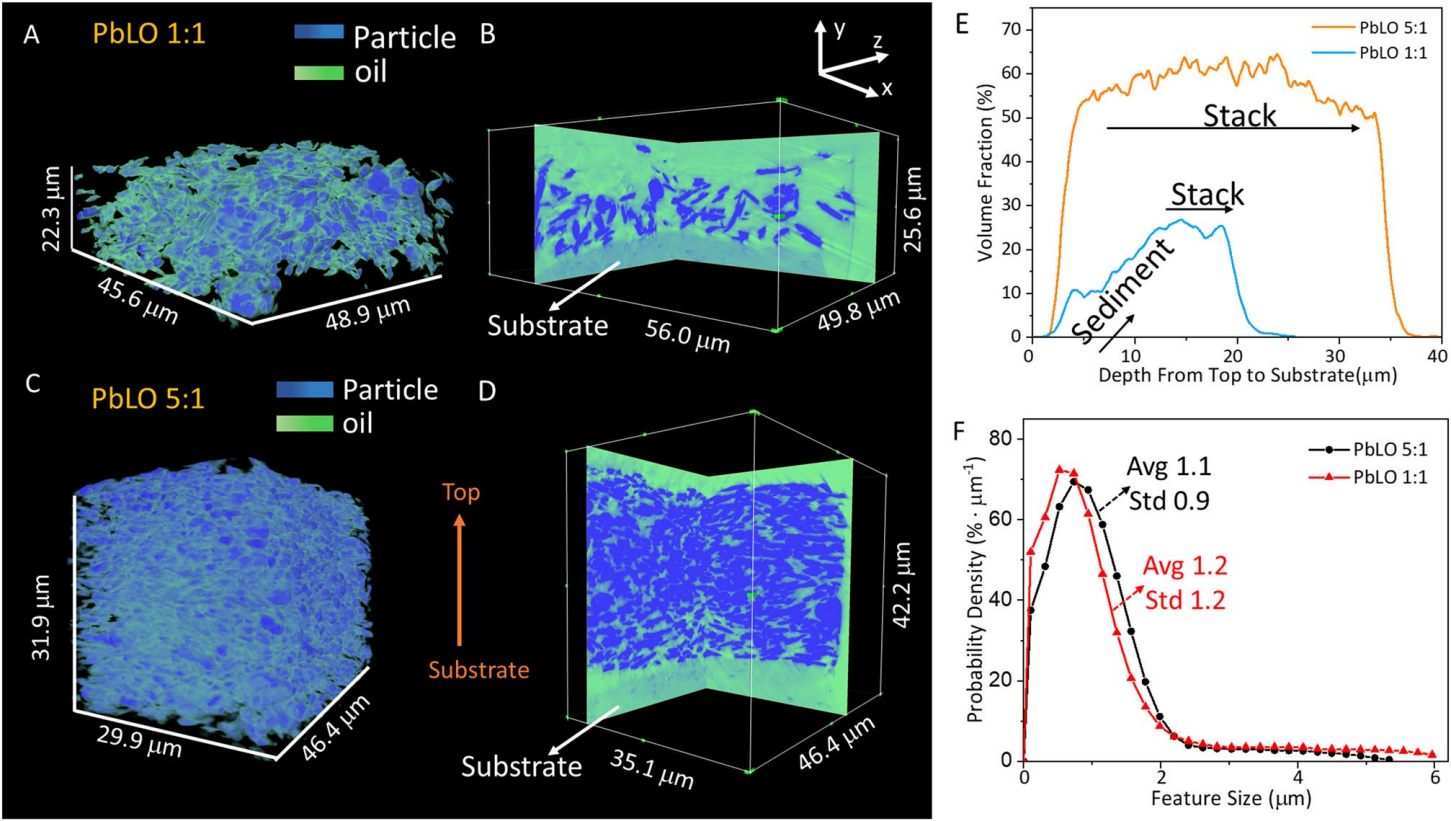
3D visualization and virtual cross-section profiles in *xy* and *yz* planes: (**A**,**B**) sample PbLO 1:1 and (**C**,**D**) sample PbLO 5:1. (**E**) Volume fraction of particles in the direction from the surface to the substrate. (**F**) Particle feature size distribution for the two PbLO paint samples.

Figure 3A–D presents the 3D morphology and the virtual cross-sections in the *xy* and *yz* planes for the PbLO samples with two different pigment to oil weight ratios, 1:1 and 5:1. The pigment particles sedimented at the bottom of the film in the sample with the larger proportion of oil (PbLO 1:1, Fig. 3A–B), while the Pb white particles stacked homogeneously in the paint film with the higher pigment proportion (Fig. 3C–D). The film thicknesses after curing were 18.3 µm for the PbLO 1:1 sample and 30.9 µm for PbLO 5:1 sample. The change in the film thickness was calculated as the difference between the wet and the dry film thicknesses determined by unilateral NMR (see, Supporting Information file), since it is not possible to study wet paint samples by nano-tomography. The thickness difference between the dry 1:1 and 5:1 PbLO samples can be explained by the fact that the volume changes in oil paints upon polymerization/drying are mainly due to the change in the volume of the organic binder.

Figure 3E presents the volume fraction of the pigment particles along the surface-to-substrate direction. For PbLO 1:1, the volume fraction of the pigment particles increases to a value of ~ 25.0% at approximately 12 μm below the surface and then it remains approximately constant from this point to the substrate. The sedimentation of the particles could potentially occur slowly during curing of the viscous oil medium if the oil content is high enough. In contrast, for PbLO 5:1, no significant change is observed for the volume fraction of the particles along the thickness direction. The particle feature size distribution was established by quantifying the probability to find various distances from the mass center to the edge of the particle. The average particle size was then calculated based on the particle size distribution by the method developed by Munch and Holzer described elsewhere^63^. The feature size distributions for the Pb white pigment particles are similar for the two samples studied, with average values of 1.2 µm and 1.1 µm for PbLO 1:1 and 5:1, respectively (Fig. 3F). This similarity in the feature size distributions indicates that the particles do not aggregate when the pigment to oil weight ratio increases. The feature size calculation for the binding medium of these two samples are shown in Figure S9 (Supporting Information file).

Because the X-ray attenuation is similar for the pores and the oil phase at the tens of nm scale, empty pores cannot be differentiated from spaces occupied by the oil phase in these experiments. Nevertheless, studies in model paint systems have proposed that the transport of water, organic solvents and other species may also take place in the oil phase^8,9^.

**Figure 4:**
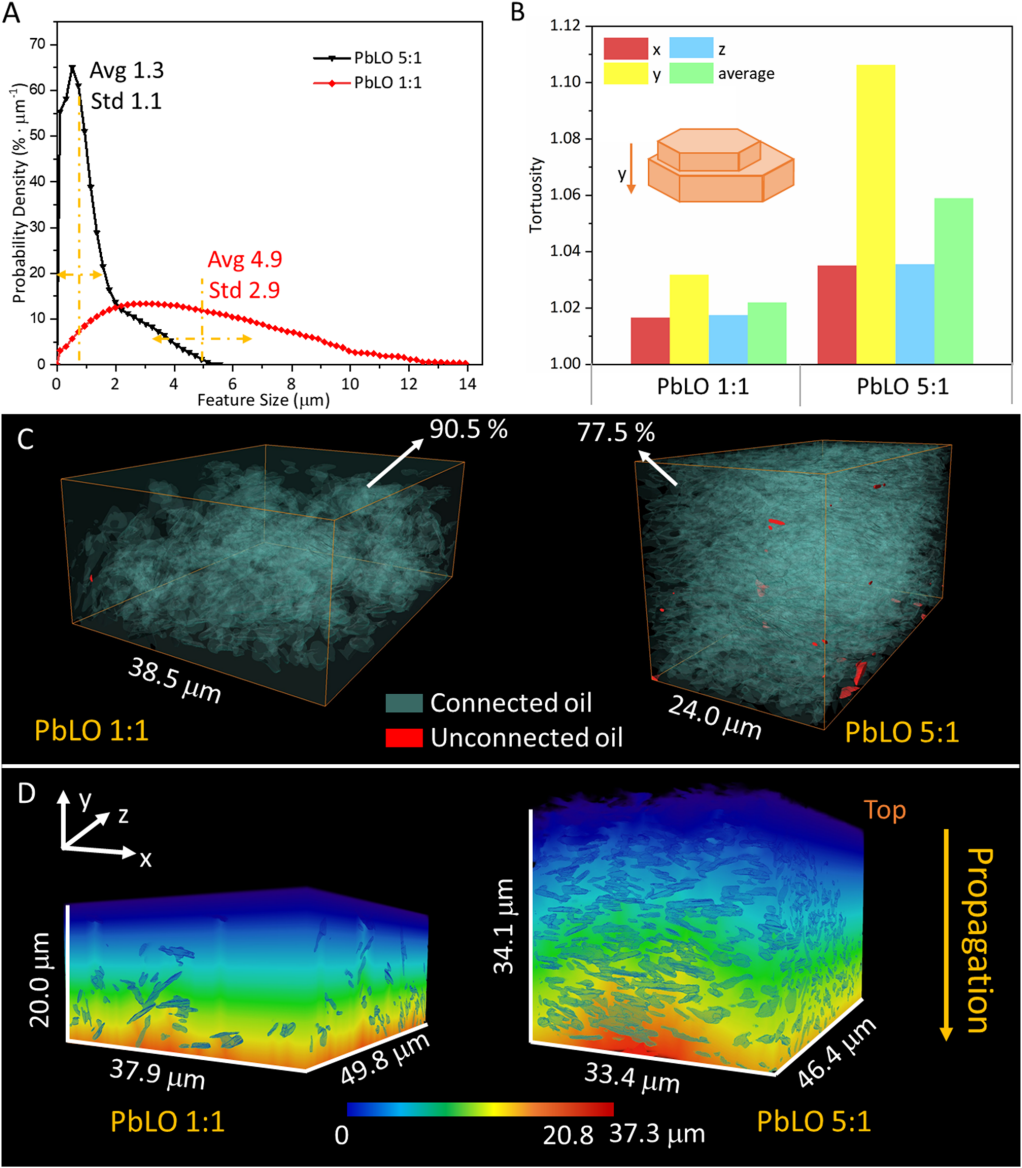
(**A**) Oil phase feature size distributions calculated for PbLO 1:1 and 5:1. (**B**) Tortuosity calculated for these two samples using the quasi-Euclidean method as the neighboring definition in the *x*, *y*, and *z* directions. (**C**) Visualization of connected and unconnected oil phase. The ratio of connected oil is 90.5% for PbLO 1:1 and 77.5% for PbLO 5:1. (**D**) Distance maps calculated by using *y* as the propagation direction.

Figure 4A–D shows the feature size distribution, tortuosity, and diffusion distance maps for the oil phase. The average feature sizes calculated for PbLO 1:1 and PbLO 5:1 samples are 4.9 µm and 1.3 µm, respectively (Fig. 4A) which means that when the weight fraction of pigment particles in the paint increases, the size distribution of the oil media decreases.

The tortuosity calculated using the quasi-Euclidean method as the neighboring definition^64^ is presented in Fig. 4B. The tortuosity inversely correlates with the average oil phase size and is larger for the PbLO 5:1 sample in all three directions, *x*, *y* and *z*. As shown above, in the PbLO 1:1 sample, the pigment particles sedimented to the bottom of the film. Thus, at the top of the paint film, the size of the oil phase is larger and the diffusion path is closer to a straight path. In the PbLO 5:1 sample, the diffusion path is more tortuous because of the higher weight fraction of pigment particles. In this sample, the particles are stacked along the vertical (y) direction, leading to a larger tortuosity in the *y* direction, and a similar tortuosity value in the *x* and *z* directions. This particle stacking is related to the pigment-to-oil ratio as discussed above, but also due to the pigment particle shape. 2PbCO_3_ $\bullet$ Pb(OH)_2_ belongs to the rhombohedral system (space group R $\stackrel{-}{3}m$), with lattice parameters $a=5.179\mathrm{ \AA }$, b = $8.492\mathrm{ \AA }$ and c = 23.702 $\mathrm{\AA }$, and a particle shape that tends to be an hexagonal platelet^65,66^. Figure 1B presents the morphology of the Pb white pigment particles and the inset in Fig. 4B shows that the particle dimension is smaller in the *y* direction than in the *x* and *z* directions so the particles can stack along the *y* direction. It is expected that a decrease in the tortuosity of a paint film will result in an increase in the rate of solvent penetration in a conservation treatment involving surface cleaning.

Figure 4C shows the distribution of connected and unconnected oil. In PbLO 1:1, the volume fraction of connected oil is ~ 90.5%, while the fraction of connected oil is 77.5% in PbLO 5:1. The higher fraction of connected oil indicates that a smaller amount of oil is enclosed in the structure. The unconnected oil may be at the edge of the sample or exist as isolated medium. Figure 4D presents the distance maps calculated along the vertical (*y*) direction as the propagation direction. The 3D distance maps help to visualize the tortuosity calculated based on geometric propagation showing the actual path length of the oil medium. With a higher tortuosity in the *y* direction in the PbLO 5:1 sample, the diffusion path is 9.4% longer than a straight path. In contrast, the diffusion path in the PbLO 1:1 sample is only 4.0% larger than a straight path. Since the pigment particles sedimented at the bottom of the paint film in PbLO 1:1, the initial diffusion rate of the species, when moving from the surface of the paint film towards the substrate, would be faster at the beginning and would slow down as it proceeds. On the other hand, the diffusion rate for the PbLO 5:1 sample, which has a higher pigment content, would be slower overall compared with PbLO 1:1, but would remain approximately constant throughout its thickness; all this considering a pure physical process without chemical interactions.

### Effect of Al stearate on the 3D morphology, tortuosity and distance maps of zinc white oil paints

**Figure 5:**
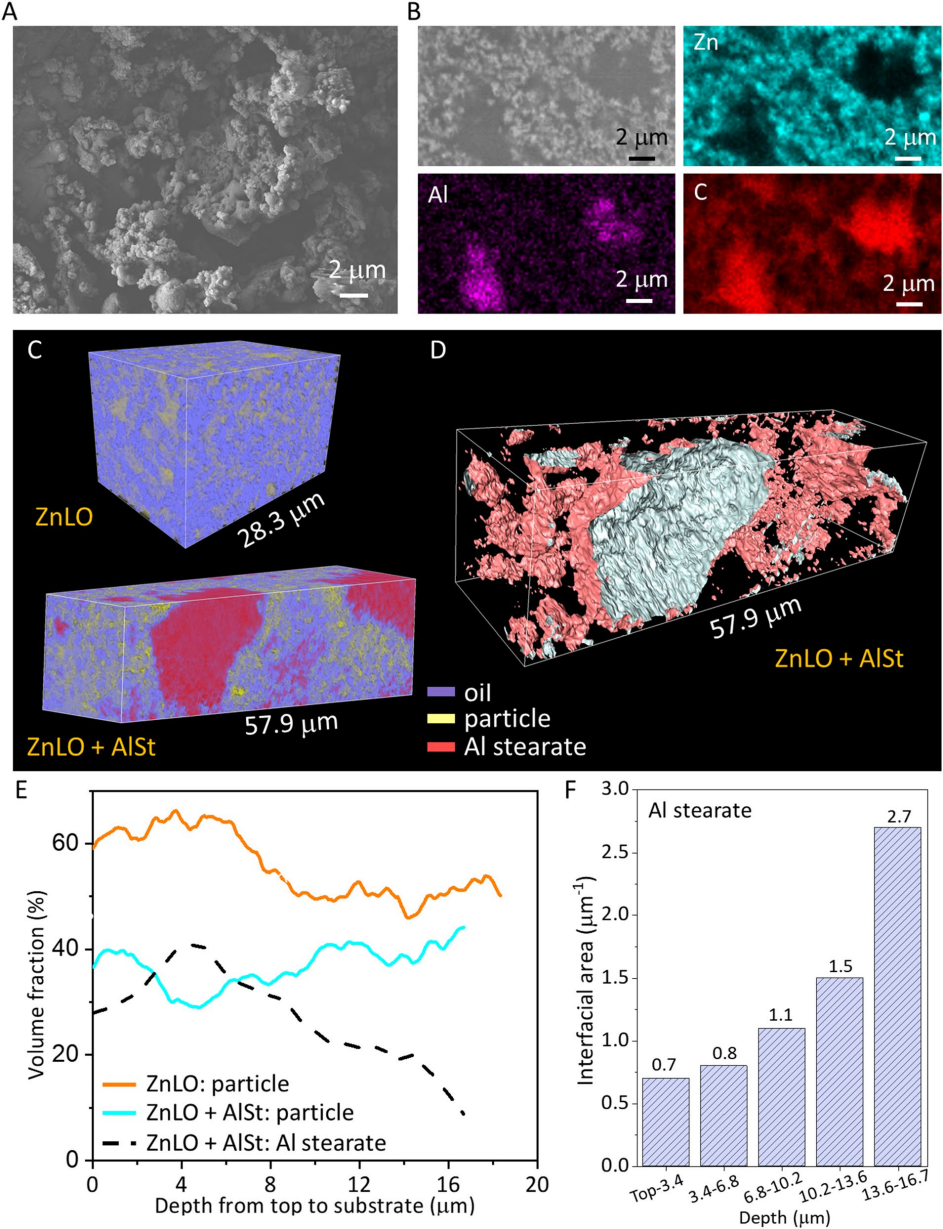
Characterization of the effect of Al stearate. (**A**) Surface morphology of Al stearate powder. (**B**) Cross-sectional image and EDS distribution maps for Zn, Al and C in the ZnLO + AlSt sample. (**C**) 3D visualization of ZnLO and ZnLO + AlSt samples by X-ray nano-tomography. (**D**) Surface structure and shape of Al stearate agglomeration. (**E**) Volume ratio of particles and Al stearate in the ZnLO and ZnLO + AlSt samples. (**F**) Interfacial area distribution of Al stearate agglomerates at different depths.

It has been demonstrated that, in oil, nano-sized micelles of Al soap aggregate into jammed networks; however, over time, configuration energy is minimized by the rearrangement of the particles into more closely packed forms^47,67^. The morphology of the Al stearate powder is presented in Fig. 5A. As shown in the cross-sectional image in Fig. 1B, particles in the ZnLO + AlSt sample are not homogeneously distributed in the paint film as they are in the ZnLO sample. Energy-dispersive X-ray spectroscopy (EDS) mapping in ZnLO + AlSt (Fig. 5B) showed that some areas are Al-rich with no Zn, indicating that Al stearate aggregated as clusters. These Al stearate aggregates are irregular in shape (Fig. 5C,D). The two samples showed different particle distribution along the depth direction (Fig. 5E). In the ZnLO sample, the amount of pigment particles is smaller towards the bottom of the film. In the ZnLO + AlSt sample, the relative amount of pigment particles is smaller overall when compared to ZnLO because of the presence of Al stearate, as expected. Moreover, in this particular sample, the volume ratio of Al stearate increases towards the surface of the paint film in the ZnLO + AlSt sample. This can be visualized in Fig. 5C, where Al stearate agglomerates relatively larger in size are present towards the surface of the paint film.

Osmond *et al.* showed that the presence of Al stearate in zinc white oil paints affects zinc carboxylate formation and distribution, as they observed higher concentrations and more pronounced spatial separation of saturated C16 and C18 chain zinc carboxylates in the films^47,67^. Gabrieli *et al.* also reported that Al stearate promotes the formation of zinc soaps in zinc white-containing paints and that the amounts of soaps formed in the presence of this paint additive exceeds that expected based on an ion exchange mechanism^5^. These authors reported that zinc stearate soaps first form around the Al stearate agglomerates and eventually grow in the whole painting stratigraphy as irregularly shape particles. Therefore, the Al stearate-Zn white oil paint interfacial area is a parameter that should be considered when proposing a mechanism for the saponification process in these paints. Ergo, the surface areas of the Al stearate agglomerates were quantified at different depths of the paint film and normalized to a constant volume of the film (Fig. 5F). In the model samples studied, it was observed that the interfacial area of the agglomerates increases towards the bottom of the film from 0.7 to 2.7 µm^−1^. Since the Al stearate agglomerates have a larger surface area and the volume fraction of Al stearate is lower towards the bottom of the paint film, it is expected that saponification be more pronounced than towards the top of the film in these model samples.

**Figure 6:**
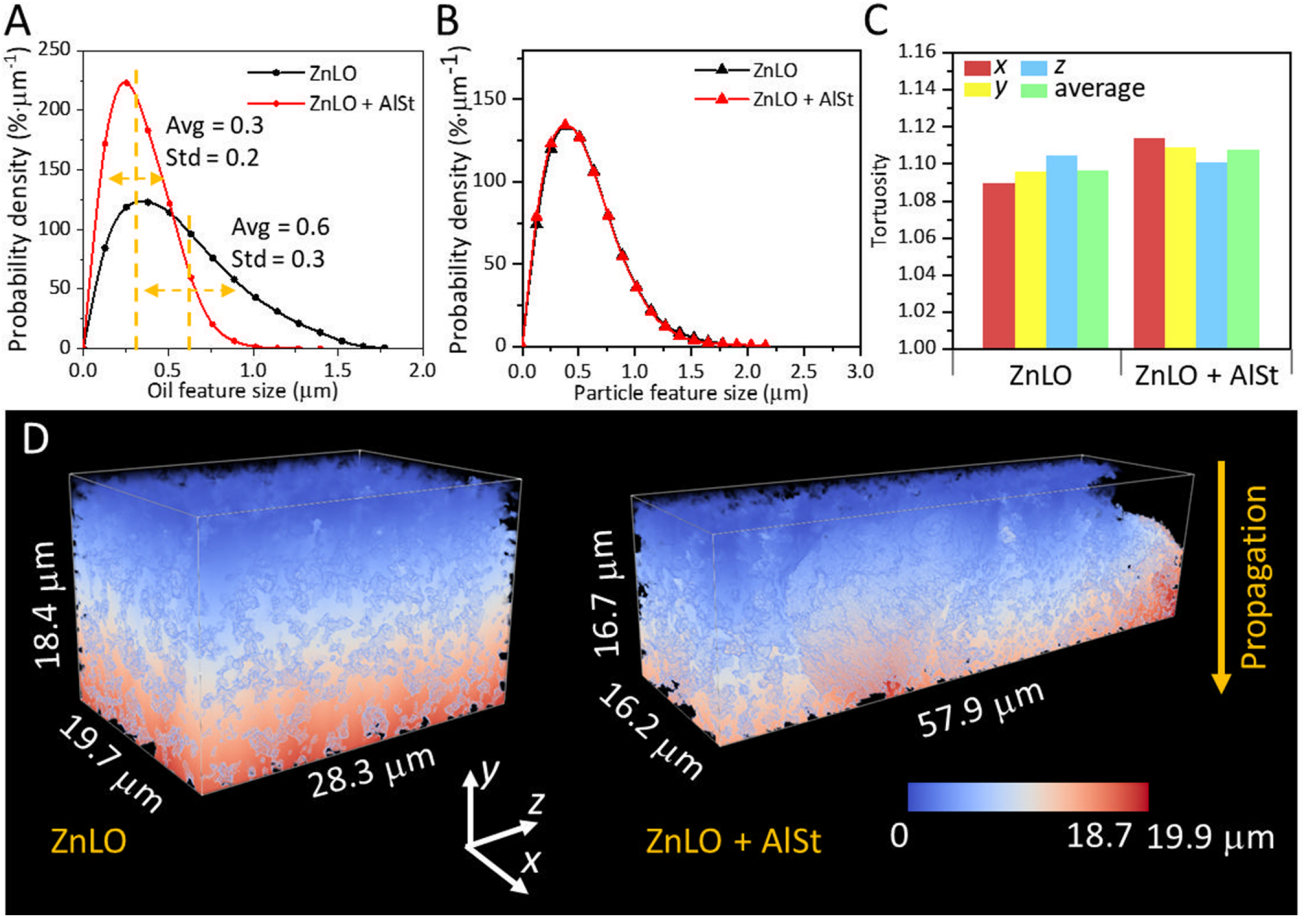
(**A**) Size distribution of the oil phase in the ZnLO and ZnLO + AlSt samples. (**B**) Size distribution of pigment particles. (**C**) Tortuosity values in *x*, *y*, *z* directions and average; *y* is the direction along the depth of the film. (**C**) Distance maps calculated using the *y* direction as the propagation direction.

Figure 6 shows the 3D morphological analysis of the ZnLO samples, with and without the Al stearate. The average size of the polymerized oil phase in the ZnLO + AlSt sample is ~ 0.3 µm which is smaller than that of the ZnLO sample at ~ 0.6 µm (Fig. 6 A). The feature size distribution of pigment particles did not show obvious differences between these two samples (Fig. 6B). Considering that X-ray nano-tomography can only resolve the agglomerated Zn white particles, not the individual particles, it may be speculated that the presence of Al stearate does not change the aggregation of the pigment particles but influences the oil curing process.

The tortuosity of the polymerized oil phase calculated along the vertical (*y*) direction for the two samples was similar at ~ 1.1 (shown for ZnLO + AlSt in Fig. 6C). The distance maps for these two samples calculated using the *y* direction are presented in Fig. 6D. The path length for the ZnLO sample is 19.9 µm, and the theoretical value is 18.4 µm. For the ZnLO + AlSt sample, the calculated length is 18.7 µm and the theoretical length is 16.7 µm. The tortuosity values along *y* direction are 1.08 and 1.12 for the ZnLO and ZnLO + AlSt samples, respectively. The tortuosity of the oil medium is the ratio between the tortuous path distance and the straight distance of the phase. The Al stearate aggregated and separated from the oil medium, so the presence of Al stearate leads to a slightly more tortuous oil matrix, which would affect the diffusion of species during the saponification process and in conservation interventions.

### Effects of the presence of an absorbing substrate on the 3D morphology

**Figure 7:**
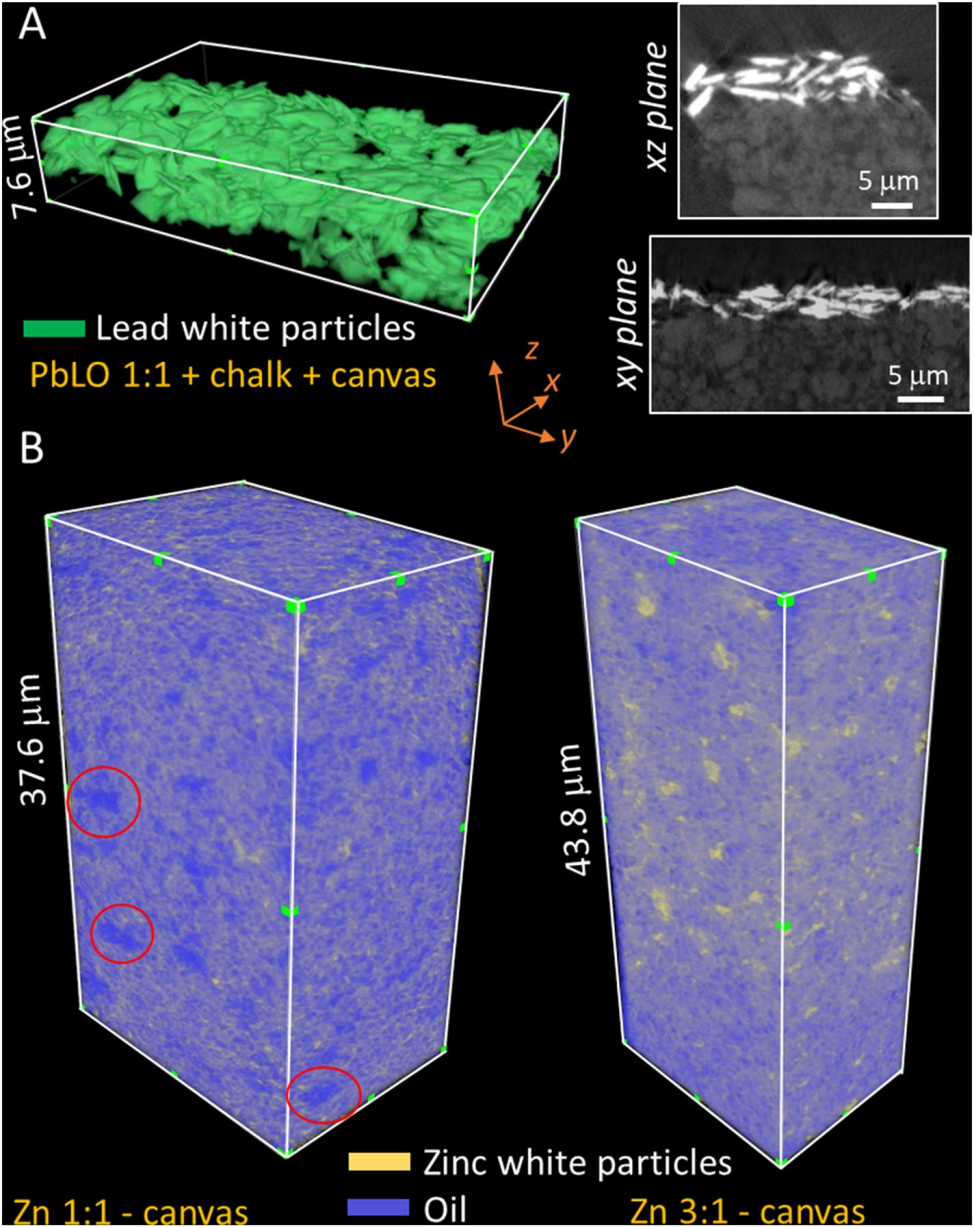
Pb white and Zn white oil paint samples applied on a canvas substrate. (**A**) 3D visualization of Pb white particles in PbLO 1:1 paint on canvas with calcium carbonate ground preparation. The virtual cross-sections on the *xz* and *xy* planes show the porous calcium carbonate layer. (**B**) 3D morphology of ZnLO paints applied on a canvas substrate with two different pigment-to-oil ratios. Pockets of oil phase in the sample ZnLO 1:1-canvas are indicated by red circles. The ZnLO 3:1-canvas sample showed a more homogeneous distribution of particles and oil phase.

The 3D morphology of a paint layer in an artistic oil painting will depend on the stratigraphy of the paint passage. For example, the presence of a porous substrate which may absorb the oil binder will have an impact on the morphology of the paint films. The 3D morphology of a PbLO 1:1 sample applied on a canvas substrate with a calcium carbonate ground preparation, presented in Fig. 7A, shows that the Pb white pigment particles are relatively more densely packed when compared to the PbLO 1:1 sample applied on Al foil (Fig. 3A). The loose network of the calcium carbonate ground, which presumably consists of empty pores and pores filled with oil, can be visualized in Fig. 7A.

A PbLO 1:1 sample deposited on a glass slide with an initial wet thickness of 177 ± 25 μm, as measured by unilateral NMR, has a 118 ± 25 μm dry thickness, a 33% change (Supplementary Information Table S3). The thickness of the PbLO 1:1 dry sample applied on canvas measured by X-ray nano-tomography is 6.3 ± 1.0 μm, which amounts to a ~ 96.4% shrinkage. Assuming that the initial wet thickness is consistent for both samples, the significantly larger shrinkage for the sample on canvas may be explained by the presence of the underlying calcium carbonate ground and canvas which absorb the oil binder.

To characterize the influence of the pigment-to-oil ratio on the morphology of oil paints, Zn white paint samples on canvas with pigment-to-oil ratios at 1:1 and 3:1 were compared (Table 1). In Fig. 7B, relatively larger oil pockets can be visualized for the sample with the smaller ratio, while the sample with the larger ratio shows a relatively more homogeneous distribution of oil and pigment particles. Therefore, the different pigment-to-oil ratios give rise to differences in the 3D morphology of the paint materials, which is expected to have a bearing on the diffusion of different compounds, such as organic solvents used in conservation treatments.

## Conclusion

In this work, reactive porous paint films consisting of inorganic pigments and a binding oil were investigated from the nano- to the microscale, to quantify their 3D morphology relevant to transport properties. Parameters associated with the 3D morphology of lead white and zinc white oil paints, such as the pigment and oil phase volume fractions, feature size distributions, tortuosity, connectivity of the oil/pore network, diffusion paths, and open porosity available for the diffusion of water, were calculated using a combination of synchrotron X-ray nano-tomography and unilateral NMR in model paint samples. The influence of the pigment type, the presence of the aluminum stearate additive, the pigment-to-oil binder ratio, and the presence of an absorbing substrate were found to have various effects on the 3D morphology of the paints. All these parameters are crucial for establishing reliable models that can predict transport properties of water and organic solvents used in conservation treatments and of species involved in deterioration reactions, such soap formation. Because the X-ray attenuation is similar for the pores and the oil phase at the tens of nm scale, it was not possible to differentiate empty pores from spaces filled by the oil phase by synchrotron X-ray nano-tomography; however, the porosity available for the diffusion of solvents, water in our case, may be measured using unilateral NMR. In cases where relatively smaller pores need to be probed, measurements by complementary techniques, such as Brunauer–Emmett–Teller (BET), are necessary. The tortuosity in the direction from the surface to the bottom of the paint films was calculated for the model samples studied, which is an important step for evaluating the effects of solvent-induced paint swelling on transport. Overall, the understanding of materials’ 3D morphology related to transport properties is important for connecting material structures to the deterioration processes and functionalities, which are also relevant to a wider range of composite materials.

## Supplementary information

Supplementary Information
