# Supplementary material for: Nano- to microscale three-dimensional morphology relevant to transport properties in reactive porous composite paint films
Source: Sci Rep. 2020 Oct 27;10:18320. doi: 10.1038/s41598-020-75040-6 (PMC7591493; doi:10.1038/s41598-020-75040-6)
Supplement: Supplementary file 1 — Supplementary Information [file 41598_2020_75040_MOESM1_ESM.docx]

***Supporting Information***

**Nano- to Microscale Three-Dimensional Morphology Relevant to Transport Properties in Reactive Porous Composite Paint Films**

Xiaoyang Liu^a,#^, Valeria Di Tullio^b,c,#^, Yu-Chung Lin^a,#^, Vincent De Andrade^d^, Chonghang Zhao^a^, Cheng-Hung Lin^a^, Molly Wagner^f^, Nicholas Zumbulyadis^e^, Cecil Dybowski^f^, Silvia A. Centeno^b,*^, Yu-chen Karen Chen-Wiegart^a,g,*^

^a^ Department of Materials Science and Chemical Engineering, Stony Brook University, Stony Brook, NY 11794, USA

^b^ Department of Scientific Research, The Metropolitan Museum of Art, NY 10028, USA

^c^ “Segre-Capitani” Magnetic Resonance Laboratory, Istituto per i Sistemi Biologi, (ISB) CNR, CNR Area Della Ricerca di Roma1, Via Salaria Km 29,300, 00015 Monterotondo, Rome, Italy

^d^ Advanced Photon Source, Argonne National Laboratory, Argonne, IL 60439, USA

^e^ Independent Researcher, Rochester, NY 14613, USA.

^f^ Department of Chemistry and Biochemistry, University of Delaware, Newark, DE 19716, USA.

^g^ National Synchrotron Light Source II, Brookhaven National Laboratory, Upton, NY 11973, USA

# These authors contributed equally to this work

* Corresponding authors:

Karen.Chen-Wiegart@stonybrook.edu, silvia.centeno@metmuseum.org

**Further details of the segmentation of the Zn white oil paint samples**

***Segmentation of the Zn white oil paint samples based on visually finding possible minimum (min), mean, and maximum (max) threshold values to account for the thresholding uncertainties.***


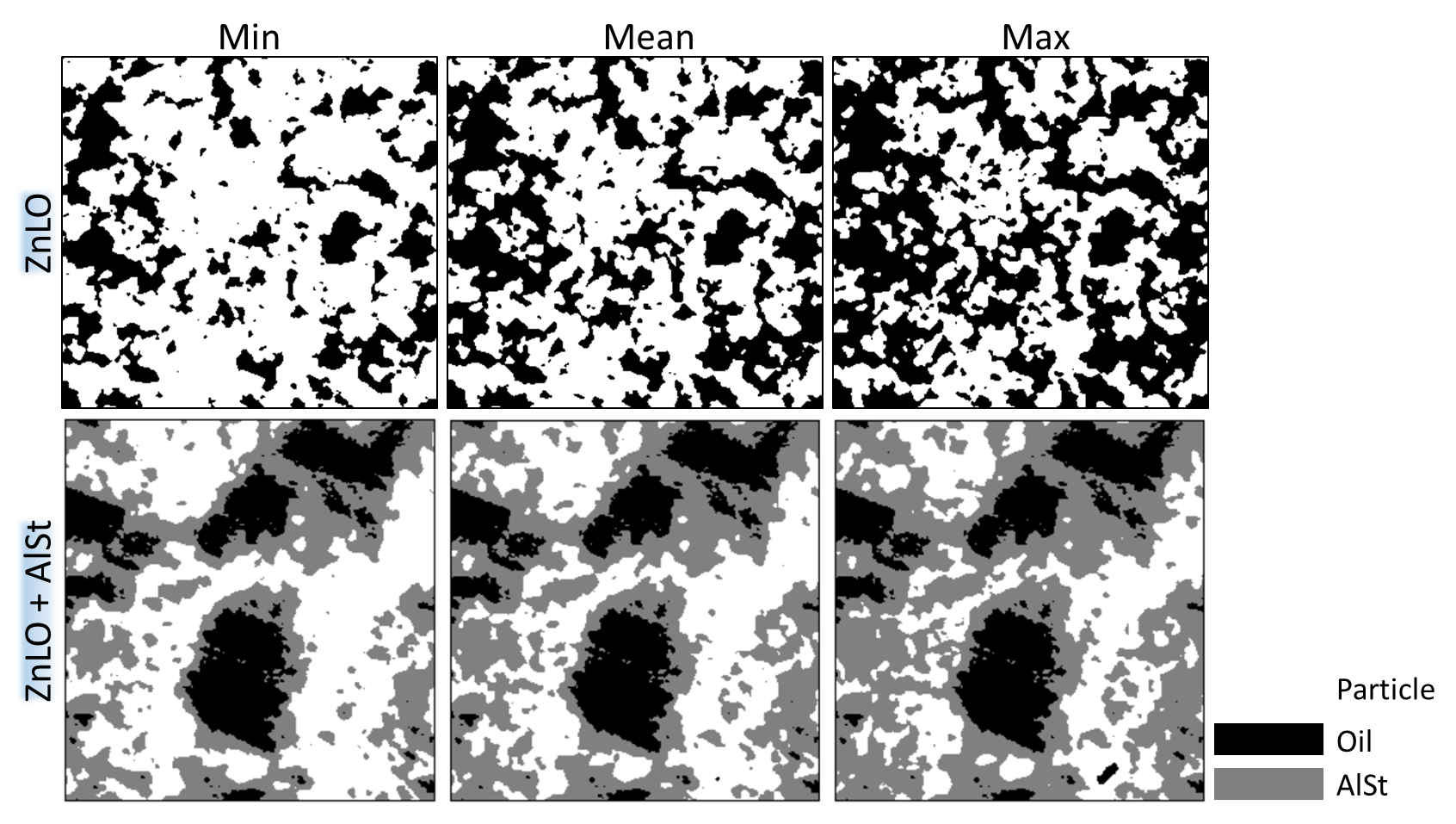


**Figure S1.** The same slices of 3D image stacks for the ZnLO and ZnLO + AlSt samples were segmented using three different threshold values. The threshold values influenced the quantification analysis of the oil and pigment phases. The min and max thresholding values provide the thresholding error bar in the quantification. In the reconstructed image stack, to remove the influence from the background, an internal part of the sample was cropped. Then, by visually finding the threshold values (min, mean, and max), the reconstructed image stack was segmented to obtain the binary images and to calculate the volume fraction, feature size and tortuosity. When the results based on different threshold values are compared, a similar trend is observed and the artifacts introduced by the segmentation can be evaluated.


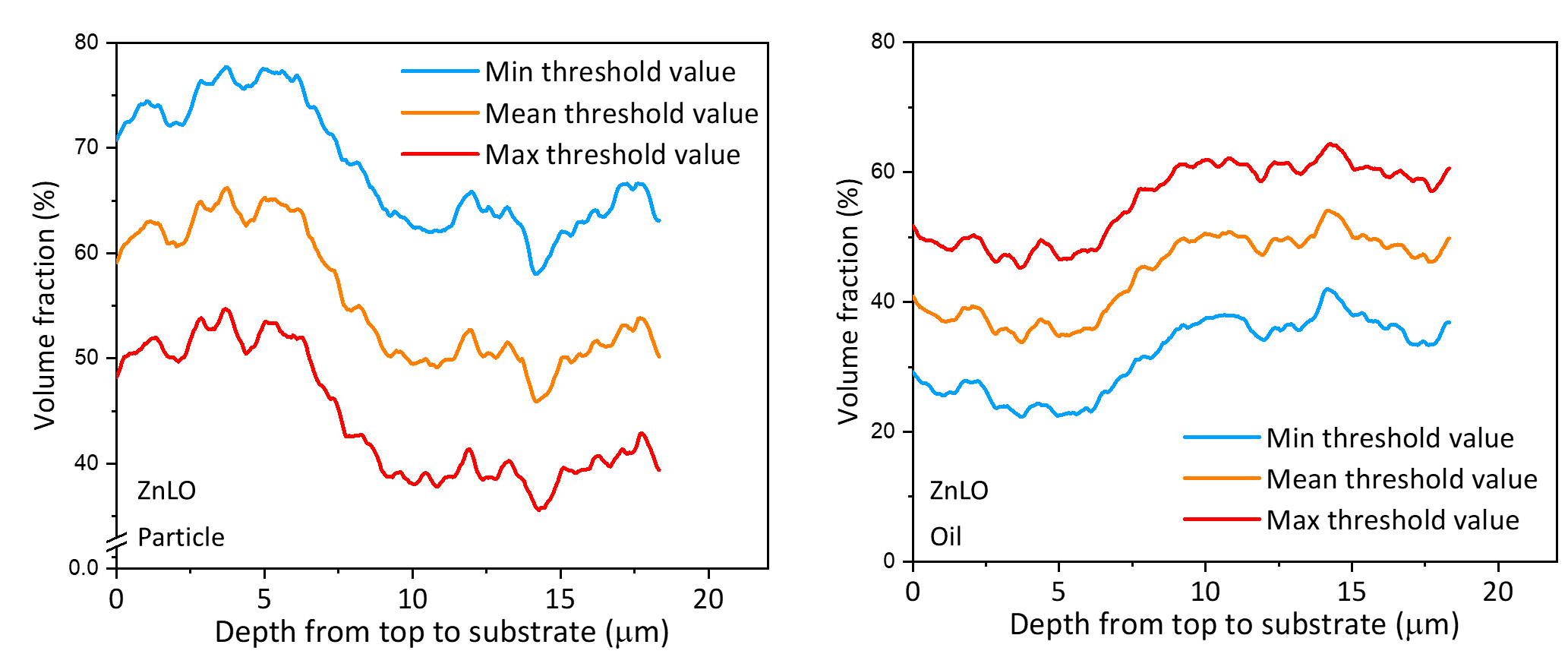


**Figure S2.** Change of the pigment and oil volume fractions with depth, from the top to the substrate, for the ZnLO sample calculated with three different (min, mean, and max) threshold values. A similar trend for the volume fractions is observed when using different threshold values.

***Pigment particle and oil phase size distributions for the ZnLO sample based on min, mean and max threhold values used in the segmentation***


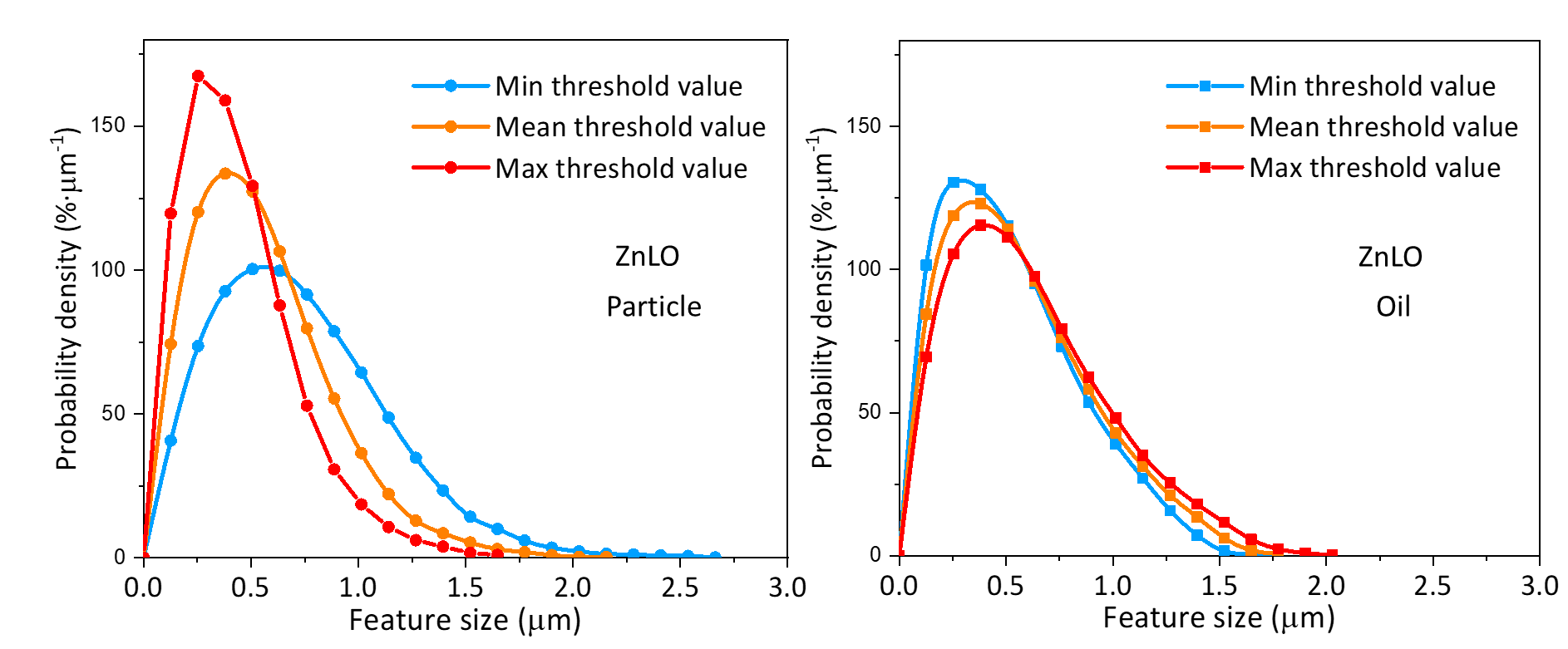


**Figure S3.** Pigment particle and oil phase size distribution for the ZnLO sample with three different (min, mean, and max) threshold values. The averages and standard deviations are presented in Table S1.

| **Phase** | **Threshold value** | **Average (mm)** | **Standard deviation** |
| --- | --- | --- | --- |
| **Pigment** | Min | 0.7 | 0.4 |
|  | Mean | 0.6 | 0.3 |
|  | Max | 0.5 | 0.3 |
| **Oil** | Min | 0.5 | 0.3 |
|  | Mean | 0.6 | 0.3 |
|  | Max | 0.6 | 0.4 |

**Table S1** Average and standard deviations for the particle and oil phase size distributions corresponding to the ZnLO sample.


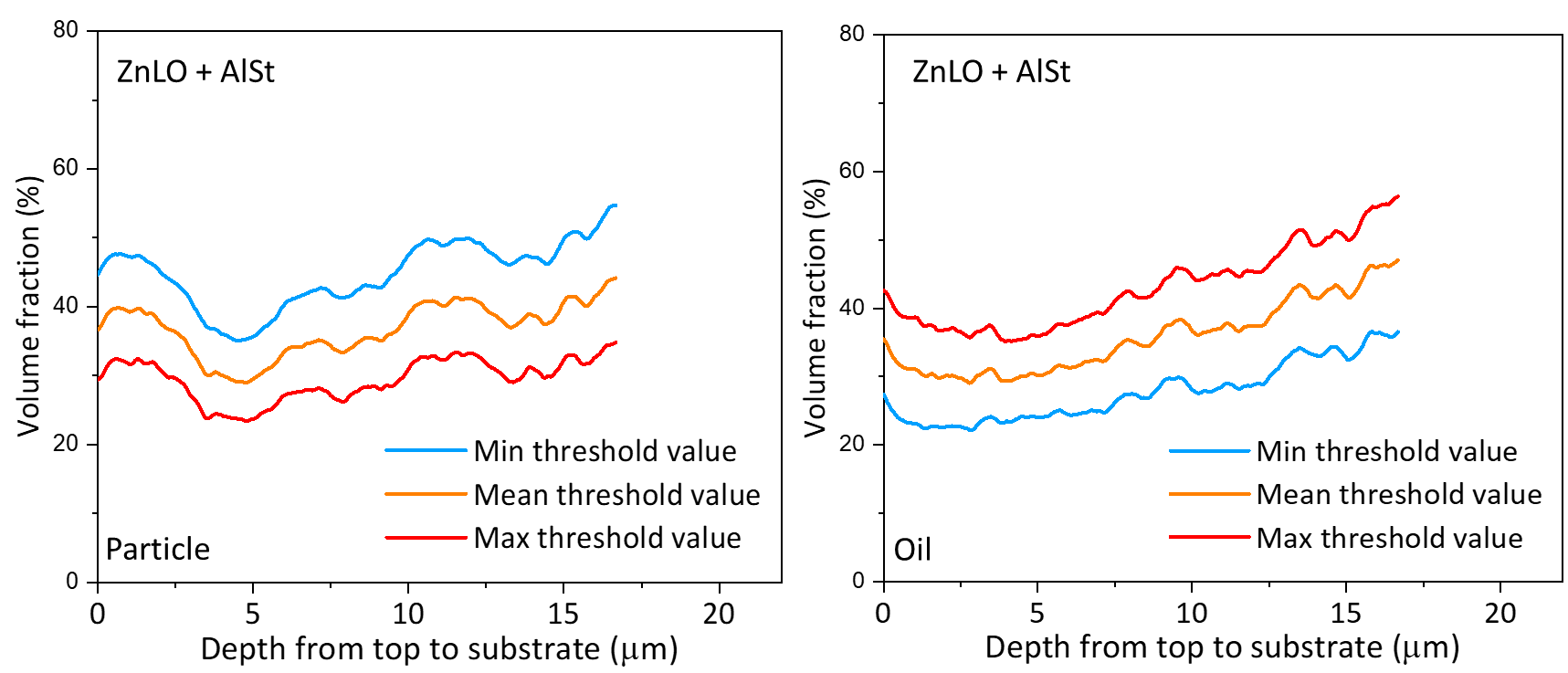


**Figure S4.** Pigment and oil phase volume fractions for the ZnLO + AlSt sample calculated with different (min, mean, and max) threshold values for the segmentation.


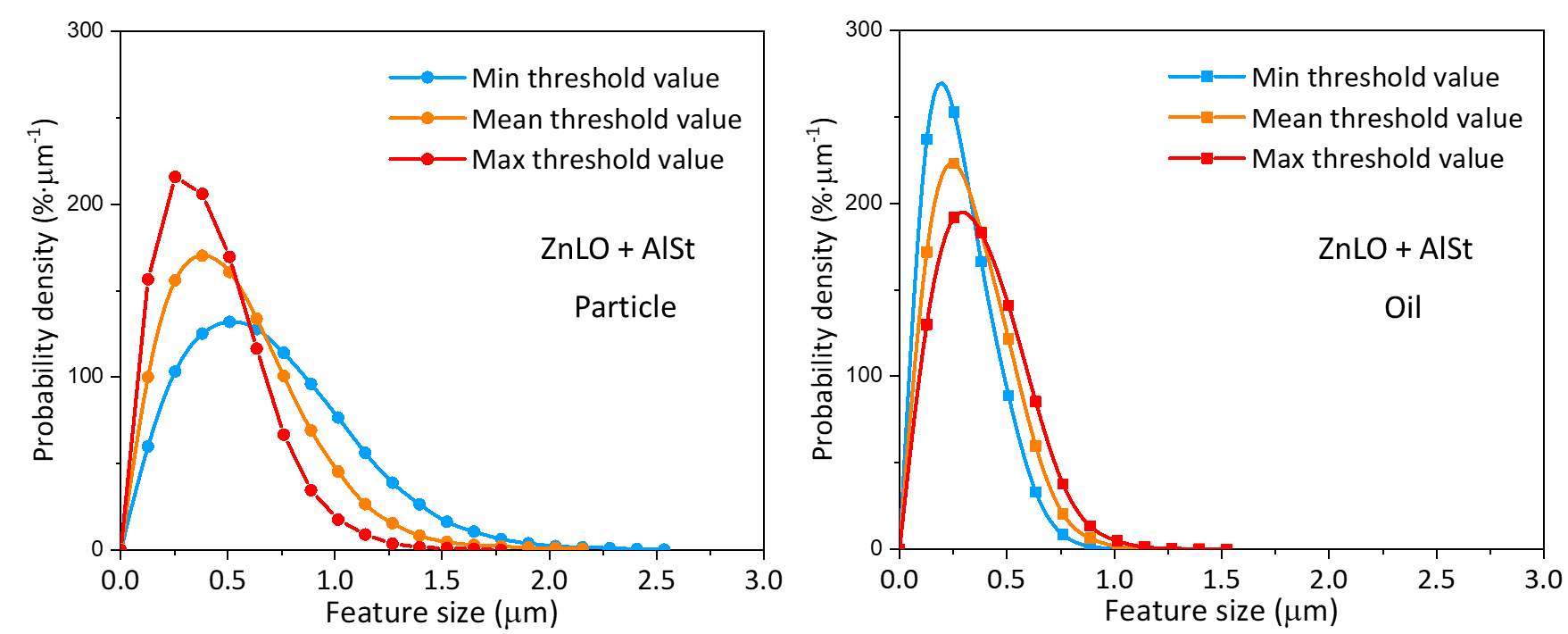


**Figure S5.** Pigment and oil phase size distributions for the ZnLO + AlSt sample calculated with three (min, mean, and max) threhold values. The averages and standard deviations are presented in Table S2.

| Phase | **Threshold value** | **Average (μm)** | **Standard deviation** |
| --- | --- | --- | --- |
| **Pigment** | Min | 0.7 | 0.4 |
|  | Mean | 0.6 | 0.3 |
|  | Max | 0.4 | 0.2 |
| **Oil** | Min | 0.3 | 0.2 |
|  | Mean | 0.3 | 0.2 |
|  | Max | 0.4 | 0.2 |

**Table S2.** Average and standard deviations for the pigment and oil phase size distributions in the ZnLO + AlSt sample.

***Changes in thickness upon curing evaluated by unilateral NMR***

The change in volume of a pure linseed oil (LO) film upon curing has been previously reported to be about 15% after 45 days of curing [1]. This change may be evaluated by weighting the film and measuring its density which involves immersing the sample in water; however, this procedure may be affected by several sources of error. As mentioned in the Introduction, unilateral NMR allows one to perform a one-dimensional measurement of the proton density as function of depth in a non-invasive manner. As linseed oil is a material rich in hydrogen nuclei, it is possible to scan the proton density along the entire thickness of a paint layer and determine changes in thickness.

In the present work, to determine the relative changes in the film thicknesses upon curing by unilateral NMR, paint films with similar compositions as those listed in Table 1 (main text) were applied on glass slides with an approximate 200 μm initial (wet) thickness. Figure S6 shows the ^1^H NMR depth profiles collected in the PbLO and ZnLO samples before and after curing for five months.


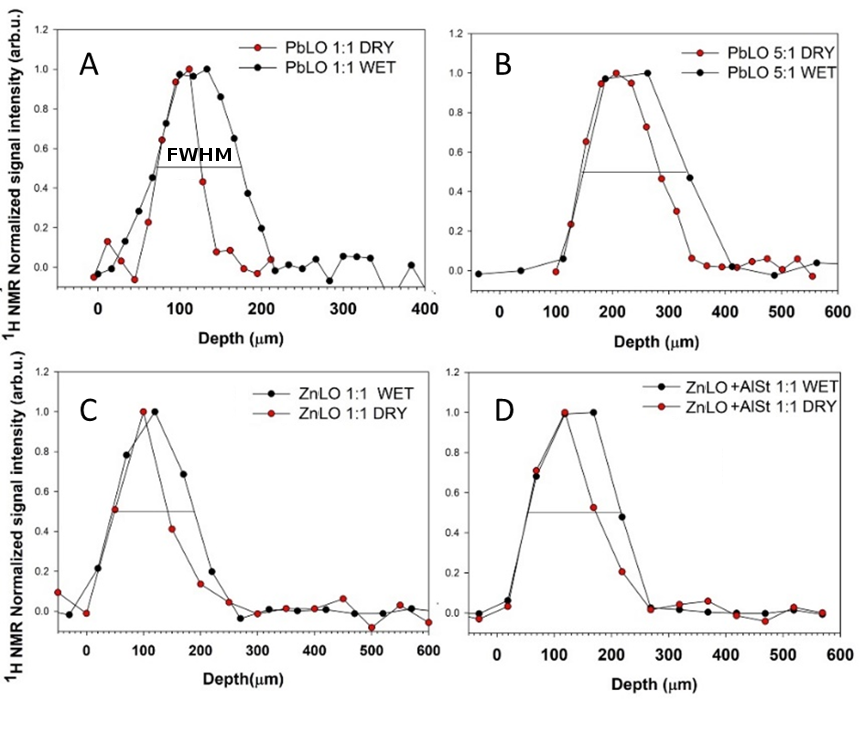


**Figure S6.** ^1^H NMR depth profiles measured in the fresh (wet) samples and after curing for five months at room temperature. (A-B) correspond to PbLO with pigment-to-linseed oil ratios of 1:1 and 5:1, respectively. (C-D) correspond to ZnLO with a pigment-to-linseed oil ratio of 1:1, without and with aluminum stearate (+AlSt).

The thicknesses of the layers were determined by measuring the full width at half maximum (FWHM), the distance from the position of half maximum amplitude of the ﬁrst edge to the equivalent point of the second edge [2]. The results are listed in Table S3. It should be noted that the wet thicknesses achieved with the film applicator set at 200 μm are smaller than this value, and vary from sample to sample as shown in Table S3.

| Sample ID | Pigment to oil weight ratio | Al stearate (wt. %) | Thickness of wet sample (μm) | Thickness of  dry sample (μm) | Shrinkage  (%) |
| --- | --- | --- | --- | --- | --- |
| PbLO 1:1 | 1 : 1 | 0 | 177 ± 25 | 118±25 | 33 |
| PbLO 5:1 | 5 : 1 | 0 | 180± 25 | 140± 25 | 22 |
| ZnLO | 1 : 1 | 0 | 144± 25 | 93± 25 | 35 |
| ZnLO+AlSt | 1 : 1 | 10 | 164 ± 25 | 119± 25 | 27 |

**Table S3**

***CPMG Processing and Calculation of the Open Porosity***

Figure S7 shows the Carr-Purcell-Meiboom-Gill (CPMG) decays measured for bulk water, for the dry paint samples, and for the water-saturated paint films, along with the relevant best fits (solid lines). As expected, the CPMG decay in bulk water follows a mono-exponential function while for the oil paint samples a multi-exponential trend is observed. Since the water saturated paint samples are complex materials, it is difficult to select the proper number of components as initial parameters in a multiexponential fitting. We used the number of components showed in the distribtuion of T_2_ obtained by ILT, as reported in the main text.

Knowing the number of mobility domains in the distribution, to extrapolate the proton content at t=0 in each domain, data was processed using a multiexponential function with a number of components n=4 in samples saturated with water, n=3 in dry paint samples, and n=1 in the bulk water. The values of M_0i_ and the T_2eff_i are reported in Table S4.


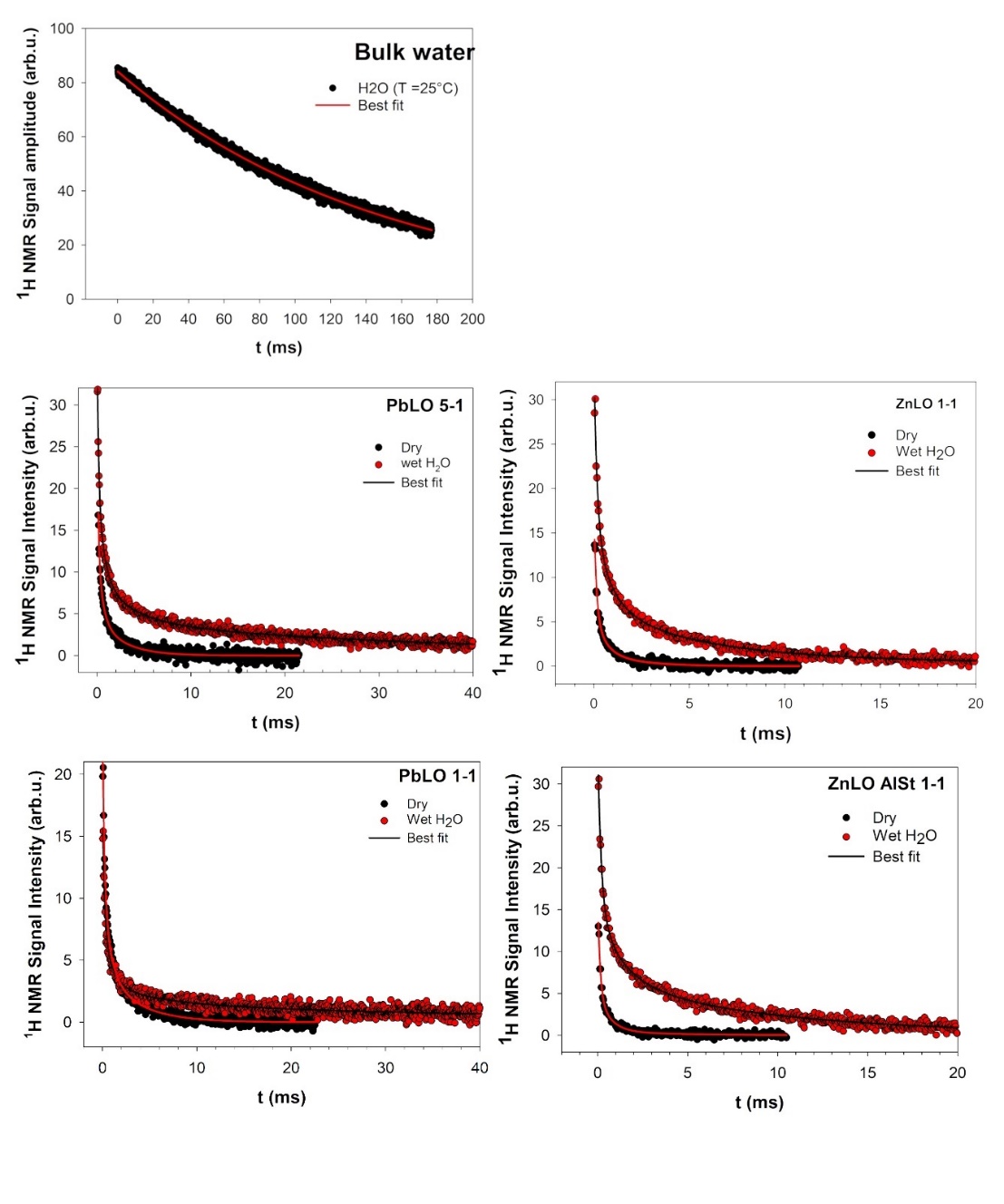


**Figure S7.** Echo envelopes obtained by applying the CPMG pulse sequence to bulk water and to the different oil paint samples before (dry) and after (wet) a 72-hour water absorption period. The best fit (solid line) was obtained by fitting a multi-exponential function.

| **Sample** | **Cured linseed oil domains*** | | | | | | **Water domain*** | | **R^2^** |
| --- | --- | --- | --- | --- | --- | --- | --- | --- | --- |
| **PbLO 5-1** | **M_0a_** ±Std.err | **T_2a_**  ±Std.err | **M_0b_**  ±Std.err | **T_2b_**  ±Std.err | **M_oc_**  ±Std.err | **T_2c_**  ±Std.err | **M_0w_**  ±Std.err | **T_2w_**  ±Std.err |  |
| Dry | 14±2 | 0.13±0.05 | 9±3 | 0.4±0.1 | 4.6±0.7 | 2.6±0.2 | - | - | 0.95 |
| Wet H_2_O | 17±1 | 0.15±0.02 | 11±1 | 0.7±0.1 | 5.3±0.6 | 3.5±0.3 | 4.1±0.1 | 36±1 | 0.97 |
|  | | | | | | | | | |
| **PbLO 1:1** | **M_0a_** | **T_2a_** | **M_0b_** | **T_2b_** | **M_oc_** | **T_2c_** | **M_0w_** | **T_2w_** |  |
| Dry | 13±1 | 0.20±0.02 | 7±1 | 0.8±0.2 | 4.1±0.5 | 3.7±0.2 | - | - | 0.98 |
| Wet H_2_O | 10±1 | 0.20±0.03 | 5±1 | 0.9±0.2 | 1.5±0.3 | 5.6±1.0 | 1.6±0.2 | 46±6 | 0.95 |
|  | | | | | | | | | |
| **ZnLO 1:1** | **M_0a_** | **T_2a_** | **M_0b_** | **T_2b_** | **M_oc_** | **T_2c_** | **M_0w_** | **T_2w_** |  |
| Dry | 12±2 | 0.12±0.02 | 4±1 | 0.5±0.2 | 1.8±0.5 | 1.9±0.6 | - | - | 0.96 |
| Wet H_2_O | 19±1 | 0.16±0.01 | 8±1 | 0.9±0.1 | 5.7±0.4 | 4.7±0.5 | 1.45±0.3 | 19±2 | 0.97 |
|  | | | | | | | | | |
| **ZnLO, Alst** **1:1** | **M_0a_** | **T_2a_** | **M_0b_** | **T_2b_** | **M_oc_** | **T_2c_** | **M_0w_** | **T_2w_** |  |
| Dry | 12±1 | 0.13±0.01 | 4±1 | 0.7±0.1 | 0.5±0.1 | 3.0±1.0 | - | - | 0.97 |
| Wet | 18±3 | 0.18±0.03 | 5±2 | 0.7±0.1 | 6.1±1 | 2.0±1.0 | 6.10±0.3 | 10.5±0.3 | 0.97 |
|  | | | | | | | | | |
| **H_2_O bulk** | - | - | - | - | - | - | 84.15±1.1 | 150±10** | 0.99 |
| *T_2_ in ms  ** the T*_2eff_* of bulk water is strongly reduced by diffusion, as unilateral NMR works with an inhomogeneous magnetic field (in homogeneous magnetic fields, T_2_ of water is typically in the order of seconds). To minimize this effect, all the measurements were performed with an echoe time (TE) as short as possible. | | | | | | | | | |

**Table S4**. Effective transverse relaxation times (T_2eff_) in all paint samples before (dry) and after water absorption (wet), obtained applying a multiexponential function.

The uncertainties associated with the M_0w_ and T_2eff_ obtained by fitting the CPMG are shown in Table S5. The measurements were repeated on three PbLO 5:1 samples saturated with water, showed in Figure S8. M_0w_ shows a variation of about 10% of the reported value. The error observed here is similar to that reported previously for measurements done with the unilateral NMR. The open porosity in PbLO 5:1 is *Θ_NMR_* = (4.7 ± 0.7)%


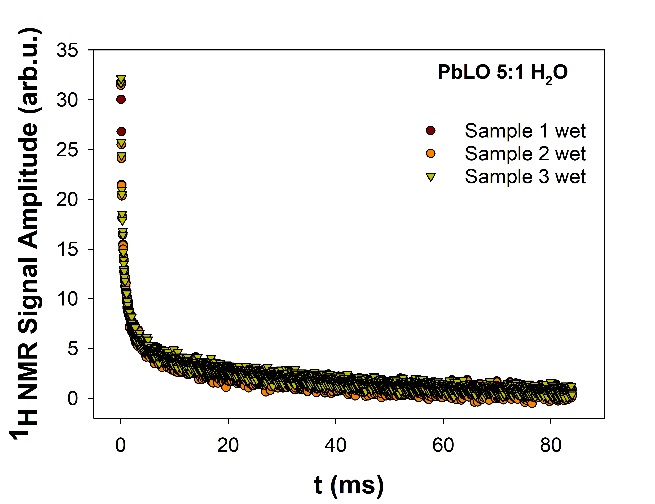


**Figure S8.** CPMG decay measured on three similar samples of PbLO 5:1 saturated with water

| **Sample** | **Cured linseed oil domains*** | | | | | | **Water domain*** | | **R^2^** |
| --- | --- | --- | --- | --- | --- | --- | --- | --- | --- |
| **PbLO 5-1** | **M_0a_** | **T_2a_** | **M_0b_** | **T_2b_** | **M_oc_** | **T_2c_** | **M_0w_** | **T_2w_** |  |
| Sample 1 | 17±1 | 0.15±0.02 | 11±1 | 0.7±0.1 | 5.3±0.6 | 3.5±0.3 | 4.1±0.1 | 36±1 | 0.97 |
| Sample 2 | 18±1 | 0.16±0.01 | 11±1 | 0.9±0.1 | 4.2±0.3 | 5.0±0.5 | 3.6±0.1 | 37±1 | 0.98 |
| Sample 3 | 21±1 | 0.20±0.01 | 9±1 | 1.0±0.1 | 2.5±0.2 | 7.4±1.0 | 4.1±0.1 | 46±1 | 0.98 |
| mean |  |  |  |  |  |  | **3.9±0.3** | **40±5** |  |

**Table S5.** Parameters obtained by a multi-exponential fitting of the CPMG decays.

**Feature size distribution of oil medium in sample PbLO 1:1 and PbLO 5:1**

**
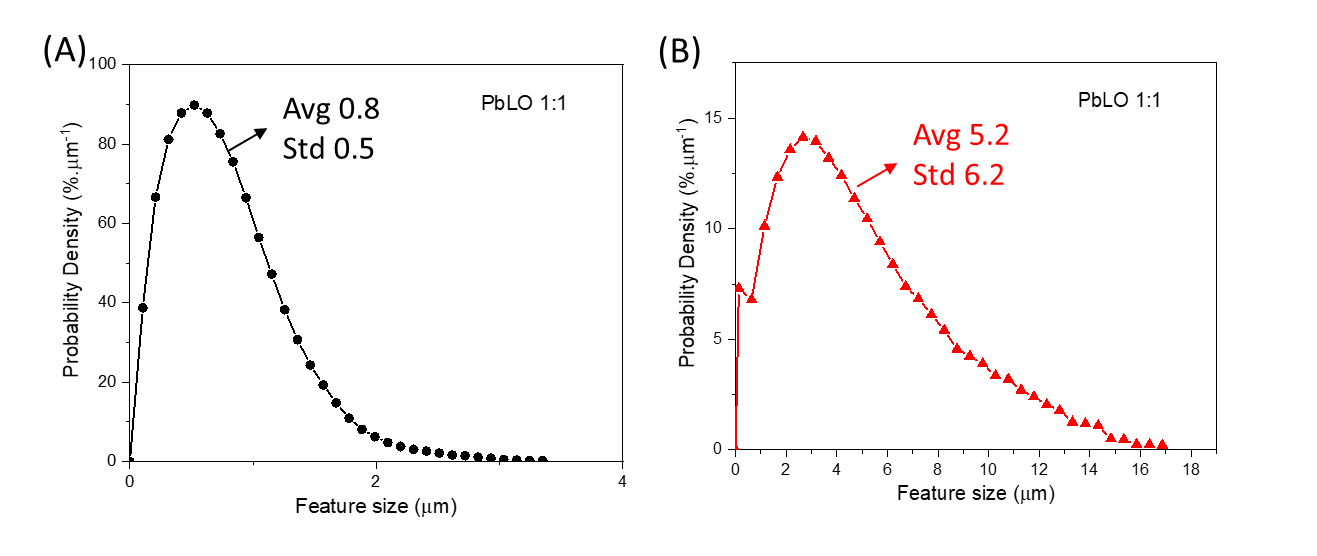
**

Figure S9. Oil medium feature size distribution with calculated average size and standard deviation: (A) PbLO 1:1; (B) PbLO 5:1

**References**

1. Svane, P., Determination of Changes in Mass and Volume of Linseed Oil During Drying. *Surface Coatings International Part B: Coatings Transactions* **2006**, *89*, 327-331.
2. Presciutti, F.; Perlo, J.; Casanova, F.; Glöggler, S.; Miliani, C.; Blümich, B.; Brunetti, B. G.; Sgamellotti, A., Noninvasive Nuclear Magnetic Resonance Profiling of Painting Layers. *Applied Physics Letters* **2008**, *93*, 033505.
